# Activation of Nucleases, PCD, and Mobilization of Reserves in the *Araucaria angustifolia* Megagametophyte During Germination

**DOI:** 10.3389/fpls.2018.01275

**Published:** 2018-08-30

**Authors:** Laura Moyano, María D. Correa, Leonardo C. Favre, Florencia S. Rodríguez, Sara Maldonado, María P. López-Fernández

**Affiliations:** ^1^Departamento de Biodiversidad y Biología Experimental, Facultad de Ciencias Exactas y Naturales, Universidad de Buenos Aires, Buenos Aires, Argentina; ^2^Consejo Nacional de Investigaciones Científicas Técnicas, Instituto de Biodiversidad y Biología Experimental y Aplicada, Buenos Aires, Argentina; ^3^Departamentos de Industrias y Departamento de Química Orgánica, Facultad de Ciencias Exactas y Naturales, Universidad de Buenos Aires, Buenos Aires, Argentina; ^4^Consejo Nacional de Investigaciones Científicas y Técnicas, Buenos Aires, Argentina

**Keywords:** *Araucaria angustifolia*, PCD, nucleases, starch, Cys-EP, megagametophyte, germination

## Abstract

The megagametophyte of mature seeds of *Araucaria angustifolia* consists of cells with thin walls, one or more nuclei, a central vacuole storing proteins, and a cytoplasm rich in amyloplasts, mitochondria and lipid bodies. In this study, we describe the process of mobilization of reserves and analyzed the dismantling of the tissue during germination, using a range of well-established markers of programmed cell death (PCD), including: morphological changes in nuclei and amyloplasts, DNA degradation, and changes in nuclease profiles. TUNEL reaction and DNA electrophoresis demonstrate that DNA fragmentation in nuclei occurs at early stages of germination, which correlates with induction of specific nucleases. The results of the present study add knowledge on the dismantling of the megagametophyte of genus *Araucaria*, a storage tissue that stores starch as the main reserve substance, as well as on the PCD pathway, by revealing new insights into the role of nucleases and the expression patterns of putative nuclease genes during germination.

## Introduction

In seeds of both Gymnosperms and Angiosperms, stored nutrients must be mobilized to support germination and early seedling growth (Young and Gallie, 2000a; Bewley et al., 2013). During germination, the main seed storage tissues, i.e., the endosperm, the perisperm, or both (in Angiosperms), and the megagametophyte (in Gymnosperms), undergo programmed cell death (PCD) In Angiosperms, the mobilization of lipids and proteins from lipid and protein bodies during germination has been studied in seeds of several species. In cereal seeds, for example, it is well documented that cells of the aleurone layer lack a central vacuole and store proteins and lipids in protein vacuoles and lipid bodies, respectively. It is also known that vacuole fusion is necessary for the establishment of the large central vacuole, which is the site where various hydrolytic enzymes and other molecules involved in PCD are localized (Zheng et al., 2017), and that damages to the integrity of the tonoplast alter the integrity of the plasma membrane, causing the collapse and subsequent death of the cell (Bethke et al., 1999). A similar process has been described in the endosperm of Dicots species such as tomato (Bewley et al., 2013), *Datura ferox* (Mella et al., 1995), and castor bean (Gietl and Schmid, 2001). However, in Gymnosperms, to date, the cell death of the megagametophyte during the mobilization of reserves is understudied and the process has been described only in *Araucaria bidwillii* (Casani et al., 2009) and *Picea glauca* (He and Kermode, 2003).

The megagametophyte of mature seeds of *Araucaria angustifolia* consists of cells with thin walls, one or more nuclei, a cytoplasm that is rich in amyloplasts, mitochondria and lipid bodies and a central vacuole that stores proteins (Panza et al., 2002). Starch is the most conspicuous reserve (Panza et al., 2002). The high water content characterizing *A. angustifolia* mature seeds (ca. 40%) is contained in the large central vacuole. Tompsett (1984) examined the relationship between seed moisture content and germination after desiccation in nine *Araucaria* species and established three moisture content groups: a group composed of *A. araucana*, *A. angustifolia*, *A. hunsteinii*, and *A. bidwillii*, which cannot be dried to below 25–40% without damage; a second group composed of *A. columnaris*, *A. rulei*, *A. nemorosa*, and *A. scopulorum*, which cannot be dried to below 12% without damage; and a third group composed of *A. cunninghamii*, which can be dried to 2% without damage. This author also found that seeds of the first group are larger and heavier and are mainly starchy, whereas those in the other groups possess mainly lipid content, and are smaller and lighter. Starchy seeds are found in species of a major clade of *Araucaria* that includes the extant sections Araucana, Bunya, and Intermedia, whereas oily seeds are found in species of the section Eutacta (Setoguchi et al., 1998).

Several reports have shown that cells undergoing PCD show the presence of some nucleases (deoxyribonucleases and ribonucleases) (Sakamoto and Takami, 2014 and references therein). To date, various plant deoxyribonucleases have been reported. Of these, several endonucleases and an exonuclease in *Arabidopsis* seem to act in leaf senescence because they were shown to be inducible at the transcript level (Sakamoto and Takami, 2014). During germination, endonucleases have been identified in the aleurone layer of cereals (Young and Gallie, 1999; Fath et al., 2000, among others) and in the embryo axes from French bean (Lambert et al., 2014).

In addition to endonucleases, KDEL-tailed cysteine endopeptidases (Cys-EPs), a group of papain-type peptidases, have been found in senescing tissue. These peptidases are synthesized as proenzymes with a C-terminal KDEL endoplasmic reticulum retention signal (Schmid et al., 1998). The signal is removed, and the enzyme separates from the endoplasmic reticulum in small vesicles called ricinosomes. Cys-EPs are able to digest extensins, which are the proteins that form the basic support for the structure of the cell wall. Cys-EPs have been detected in the endosperm of *Ricinus communis* (Schmid et al., 2001), the epigeal cotyledons of *Vigna mungo* (Toyooka et al., 2001), and the megagametophyte of *Picea glauca* (He and Kermode, 2010) during germination, as well as in the micropylar endosperm and suspensor of *Chenopodium quinoa* during seed development (López-Fernández and Maldonado, 2013b).

In the present study, we assessed the PCD of the megagametophyte of *A. angustifolia* seeds during germination, with the objective to evaluate the expression and activity of nucleases in cells that reserve starch, as well as the sequence of autophagy and PCD during the process of mobilization of reserves. After analyzing the previous reports mentioned above, we inferred that the PCD pathway of the megagametophyte of *A. angustifolia* is different from that of the aleurone layer in cereals, since, in the former, the central vacuole already exists and the main reserve is located in the plastids. The PCD pathway in *A. angustifolia* should also be different from that of the starchy endosperm of cereals (Young and Gallie, 1999, 2000b; Sabelli, 2012; Domínguez and Cejudo, 2014) and that of the starchy perisperm of quinoa (López-Fernández and Maldonado, 2013a; Burrieza et al., 2014), since, in these tissues, PCD occurs during the development of the seed and is associated with the accumulation and not with the dismantling of the reserves. It should be clarified that, in Gymnosperms, the cell death of the megagametophyte during the mobilization of reserves has been investigated in *Araucaria bidwillii* (Casani et al., 2009), a species that, like *A. angustifolia*, also produces starchy seeds. In this species, necrosis (and probably also PCD in some cells) was identified by DNA fragmentation, changes in the size and morphology of nuclei, and a substantial increase in proteolytic activities, including those of caspase-like proteases. It is also worth mentioning that, in *Araucaria araucana*, starch degradation is initiated by a-amylase and phosphorylase in the embryo and by phosphorylase mainly in the megagametophyte (Cardemil and Reinero, 1982; Cardemil and Varner, 1984).

To describe the PCD of the megagametophyte during germination of *A. angustifolia* seeds, in the present study we analyzed the mobilization of reserves at different times following imbibition, and investigated the characteristics that define the process of PCD and autophagy such as activation of Cys-EPs, nuclear fragmentation and internucleosomal DNA cleavage. Likewise, we analyzed genes of S1 nuclease-like endonucleases and *Staphylococcus* nuclease-like (SN) endonucleases and a gene with a DNase-RNase domain not classified as S1 or as Tudor because it lacks these domains.

## Materials and Methods

### Plant Material

*Araucaria angustifolia* seeds were collected from trees grown in natural populations in the Botanical Garden “Arturo E. Ragonese”-INTA Castelar, situated in Buenos Aires province, Argentina (34°40′S 58°39′W), from March to May 2017. Seeds were surface-disinfected with 5% NaClO for 15 min and then allowed to germinate onto imbibed perlite in a growth chamber under controlled conditions of 16 h light/8 h dark cycles at 25°C. At 14, 28, and 42 days after germination (DAG), specifically following radicle protrusion, the seeds were dissected, and the megagametophytes were either used fresh or milled after freeze-drying and the flours stored at -80°C until use. Experiments reported here were repeated with at least three independent biological replicates; the results were comparable across experiments, unless otherwise stated.

### Sample Preparation for Histological Analysis

Samples were collected at 0, 14, 28, and 42 DAG and prepared for microscopy according to López-Fernández and Maldonado (2013a) by fixation in 4% paraformaldehyde, 0.1 M phosphate buffered saline (PBS) pH 7.2 for 24 h at 4°C. After rinsing, the samples were dehydrated in an acetone series, and then embedded in Technovit 8100 (Kulzer and Co., Germany). Resin was polymerized at 4°C. The sections were stained with 0.5% toluidine blue O (Sigma-Aldrich, St. Louis, MO, United States) in aqueous solution, or used without staining procedure.

For the TUNEL assay, samples were fixed at 4°C in 4% paraformaldehyde (0.1 PBS; pH 7.2), dehydrated in a graded ethanol series (30, 40, 50, 60, 70, 80, 90, and 100%) and embedded in LRW resin (Polyscience, Inc., Warrington, PA, United States; 17411) as previously described by Harris et al. (1995). Semi-thin sections (1 μm thick) were mounted on glass slides. To identify starch and proteins, sections were stained with Lugol solution (Biopack 151205, Argentina) and Amido Black (Anedra 6952, Argentina), respectively (Jensen, 1962; Owens et al., 1993).

### Evans Blue Staining

Megagametophytes at 0 and 42 DAG following germination were stained with 1% Evans Blue for 1 min, destained with deionized water for 1 h, and photographed under a dissecting microscope.

### RNA Extraction and Semi-Quantitative PCR (RT-PCR)

The megagametophytes from *A. angustifolia* seeds were homogenized in liquid nitrogen with pestle and mortar, and total RNA was extracted using the protocol described by Chang et al. (1993). The quantity and purity of the RNA samples were assessed using a NanoDrop 2000 spectrophotometer (Thermo Fisher Scientific, Waltham, MA, United States); samples with 260/280 nm and 260/230 nm ratios between 1.8–2.2 and 1.6–2.2, respectively, were considered pure enough. The integrity of the samples was confirmed by electrophoresis on a 1.5% (w/v) agarose gel. Total RNA was treated with DNase I (New England Biolabs). Then, first-strand cDNA was synthesized using M-MuLV Reverse Transcriptase (New England Biolabs) and d(t) 20 oligonucleotide, following the manufacturer’s instructions.

Gene expression was evaluated through semiquantitative RT-PCR. Nuclease primers were designed using Primer3Plus Program (Untergasser et al., 2007). The endogenous normalization was performed using Ubiquitin 1 gene (Schlögl et al., 2012). The primer sequences are shown in **Table 1**. The PCR reactions were conducted in a total volume of 25 μL containing 5 μL of 1:10 diluted cDNA, 0.5 U Taq polymerase (Invitrogen), 0.2 mM dNTP, 0.1 μM for a specific sense and anti-sense primers, 5 μL 10× PCR buffer (Invitrogen) and 0.5 mM MgCl_2_. The thermal cycle conditions used were: 94°C for 3 min, 94°C for 30 s, 58°C for 30 s, 60°C for 30 s and 72°C for 1 min. The numbers of cycles were specific for each pair of primers. The PCR products had a length between 160 and 247 bp. The RT-PCR products were resolved on 1.5% (w/v) agarose gel and stained with ethidium bromide (0.5 μg/mL).

**Table 1:** Primer sequences used in this work.

Primers	Sequence	Amplified fragment
AaUb1-Fw	5′GTCGGATGTGTTTCATCCTAATG3′	160 bp of the Ubiquitin 1
AaUb1-Rr	5′CTTCTGGATTTGCAGGACTTG3′	gene
Ac520-Fw	5′TAGGGCAATGGTGGTTAATG3′	229 bp of an
Ac520-Rv	5′AAATTCTGCTGCCTCATGTC3′	uncharacterized protein
		(endonuclease activity)
Ac114-Fw	5′AGTGCATGAGGCTTACCTTG3′	218 bp of an
Ac114-Rv	5′TAACCATTCCCGAACAAGAG3′	uncharacterized protein
		(endonuclease activity)
Ac343-Fw	5′GAGATGAAGGTGGAAACACG3′	247 bp of an
Ac343-Rv	5′AGACGAATGCTTTCAGTTGC3′	uncharacterized protein
		(endonuclease activity)


### *In-gel* Nuclease Activity Assays

For *in-gel* nuclease assays, megagametophytes at different stages were ground in liquid nitrogen and homogenized in extraction buffer [10 mM Tris-HCl pH 8.0, 1 mM EDTA, 0.1 (w/v) % SDS, 0.1 phenylmethylsulphonyl fluoride (PMSF) from Roche (Mannheim, Germany), and 1 mM dithiothreitol (DTT)]. Equal amounts of protein (15 μg) were incubated for 20 min at 40°C in buffer [0.125 M Tris pH 6.8, 10% (v/v) glycerol, 2% (w/v) SDS, 0.01% (w/v) bromophenol blue] and resolved on 12% SDS-PAGE gels containing 0.3 mg mL^-1^ herring sperm DNA (Biodynamics, Argentina). For single-stranded DNase activity, DNA was boiled for 5 min immediately prior to pouring the gel. The gels were soaked in 25% 2-propanol and 1 mM EDTA for 15 min to remove SDS as previously reported by Leśniewicz et al. (2010). Subsequently, the gels were incubated overnight in 25 mM sodium acetate-acetic acid buffer [pH 5.5, 0.2 mM DTT and 1% (v/v) Triton X-100] or 10 mM Tris–HCl neutral buffer [pH 8.0, 0.2 mM DTT and 1% (v/v) Triton X-100] at 37°C. *In-gel* assays in the presence of cations were performed as above, in buffer containing 0.1 mM ZnSO_4_ or 10 mM CaCl_2_. After incubations, the gels were washed for 5 min in cold stop buffer [10 mM Tris–HCl (pH 8.0), 1 mM EDTA]. Nuclease activity was detected as a negatively stained band revealed by staining the gels with 0.01 mg/mL ethidium bromide and photographed using the Box GeneSnap software from Syngene. The band intensity was analyzed using the Gel-Pro Analyzer Software (Media Cybernetics Inc.). All SDS-PAGE results were replicated a minimum of three times.

### DNA Isolation and Fragmentation Analysis

Genomic DNA was isolated by the cetyl-trimethyl-ammonium-bromide (CTAB) method (Doyle, 1991). Then, 200 mg of three different megagametophytes were ground with liquid nitrogen into a fine powder and mixed with 400 μL CTAB solution [1.4 M NaCl; 2% (w/v) PVPPM_40,000_, 20 mM EDTA (pH 8.0), 100 mM Tris-HCl, pH 8.0; 2% (w/v) CTAB]. The mix was incubated for 15 min at 70°C. An equal volume of chloroform:isoamyl alcohol mixture (24:1) was added and, after shaking gently, the mixture was centrifuged for 10 min at 10,000 g. The upper aqueous phase was removed and the total DNA was precipitated by addition of 700 μL 70% (v/v) ethanol. DNA was recovered by centrifugation for 2 min at 10,000 g. The yield and quality of the DNA obtained were assessed in a NanoDrop 2000 spectrophotometer (Thermo Fisher Scientific, Waltham, MA, United States). For DNA-fragmentation analysis, 20 μg of each sample was separated on a 2% (w/v) agarose gel and stained with ethidium bromide (0.5 μg/mL). A Thermo Fisher Scientific^TM^ DNA GeneRuler^TM^ 100 bp was used as a reference.

### Protein Concentration

The protein concentrations were determined as described by Bradford (1976), using a Quick Start Bradford Protein Assay Kit 1 (500–0201; Bio-Rad, United States Laboratories) and bovine serum albumin (BSA) as a standard (Bio-Rad Laboratories, United States).

### TUNEL Assay on Megagametophyte Cells After Germination

Nuclear DNA fragmentation was detected by TdT-mediated dUTP nick-end labeling (TUNEL) according to the protocol provided by the manufacturer (*In situ* cell detection kit TMR red, Roche, Merck KGaA, Darmstadt, Germany). Briefly, tissue sections from proximal to embryo area were treated with 0.05% Tween 20 in PBS for 15 min at room temperature, to facilitate penetration of the labeling reagents. The slides were incubated in the TUNEL reaction mix at 37°C for 60 min. To prepare negative controls, sections were incubated without the Terminal deoxynucleotide Transferase (TdT) enzyme from the reaction mixture; to obtain positive controls, sections were pretreated with DNase I (image not shown). The percentage of TUNEL positive nuclei, at 0, 14, 28, and 42 DAG, were calculated from 100 nuclei randomly selected, for each section. At least 5 semi thin sections of a different megagametophyte tissue were observed.

### Cys-EP Immunological Assays

#### Western Blotting

Megagametophyte tissue of *A. angustifolia* from different DAG and endosperms of *R. communis* from seeds 5 DAG were ground in liquid nitrogen and homogenized in extraction buffer [50 mM Tris -HCl (pH 8), 50 mM DTT, 1 mM EDTA and 1 mM PMSF]. The cellular extracts were centrifuged for 10 min at 14,000 g, 4°C. Western blot analyses were performed as previously described in López-Fernández and Maldonado (2013b), with minor modifications. Equal amounts of protein were separated using SDS–PAGE and electrotransferred to a PVDF membrane (Millipore Corporation, Bedford, MA, United States) at 100 V for 60 min. The membrane was immersed in 3% (w/v) BSA in a TTBS solution [0.2 M Tris-HCl (pH 7.6), 1.37 M NaCl, 0.1% (v/v) Tween-20] overnight at 4°C. The proteins were incubated with a primary antibody raised against purified 35 kDa Cys-EP (Schmid et al., 1998) diluted 1:1000 in 1% (w/v) BSA in TTBS for 2 h at room temperature and subjected to five 5 min rinses in a TTBS solution. The membrane was then incubated with a secondary alkaline phosphatase-conjugated goat anti-rabbit antibody (Sigma A3587, Merck KGaA, Darmstadt, Germany) diluted 1:5000 in TTBS for 1:30 h at room temperature. The secondary antibody was detected with NBT/BCIP (Promega, Madison, WI, United States).

#### *In situ* Immunolocalization

Immunolocalization was carried out according to the protocol described by Schmid et al. (1999) and López-Fernández and Maldonado (2013b). Briefly, after blocking with 1% (w/v) BSA in PBS for 90 min, the slides were incubated with anti-Cys-EP (dilution 1:100 in 0.1% BSA/PBS) overnight at 4°C. After washing in PBS plus 0.05% (v/v) Tween 20 (PBST) three times for 10 min, the slides were incubated with a fluorescent anti-rabbit ALEXA 488 IgG (Invitrogen, Thermo Fisher Scientific, Waltham, MA, United States) antibody applied 1:1000 in 0.1% BSA/PBS for 1 h at room temperature in the dark. After additional rinses in PBST, sections were examined by epifluorescence and light microscopy.

### Microscope Settings

Images were obtained by epifluorescence and light microscopy with an Axioskop 2 microscope (Carl Zeiss, Jena, Germany). All images were captured with an EOS 1000D camera (Canon, Tokyo, Japan), analyzed using the AxioVision 4.8.2 software package (Carl Zeiss, Jena, Germany), and compiled (Photoshop version CS6; Adobe Systems, San Jose, CA, United States). Rhodamine filters (excitation 520–560 nm, emission 570–620 nm) and DAPI filters (excitation 340–390 nm, emission 420–470 nm) were used to examine samples by TUNEL assay, whereas Alexa filters (excitation 450–490 nm, emission 515–565 nm) were used to examine samples for immunofluorescence assays.

## Results

### Histochemical Staining and Analysis of Tissue Sections Revealed Progressive Megagametophyte Degradation, Mobilization of Reserves and Cells Undergoing Programmed Cell Death (PCD) During Germination

**FIGURE 1:**
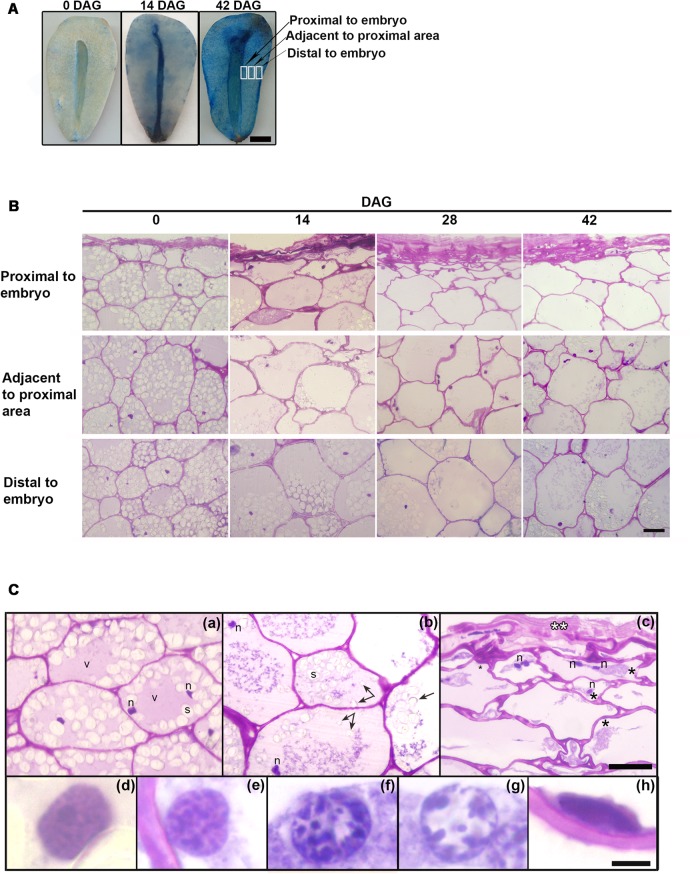
**(A)** Evans Blue staining at 0 and 42 DAG in the megagametophyte after extraction of the embryo. In each case the figure is a representative result of observation of at least 6 seeds. Scale bar = 0.5 cm **(B)** Changes occurring from the proximal to distal zones in the megagametophyte, at 0, 14, 28, and 42 DAG. Scale bar: 50 μm. **(C)** In detail, megagametophyte tissue (14 DAG). **(a)**, Distal section of the tissue; **(b,c)**, Adjacent to proximal sections; in c collapsed cell area. **(Ca,c)**, some cells show two nuclei. **(d–h)**, Progressive changes in nuclear morphology. The black asterisk indicates plasmolysis, the double white asterisk indicates crushed cells, and the black arrow indicates amyloplast discharged into central vacuole. Scale bar: **(a–c)** = 50 μm, **(d–h)** = 5 μm. Abbreviations: *n*, nucleus; *v*, vacuole; *s*, starch. Each image is a representative result of observation of at least 30 semi-thin sections of megagametophyte tissue at different DAG.

During germination, the megagametophyte changed its color progressively from white and bright to brownish. **Figure 1A** shows the progressive cell death observed in the megagametophyte, from the innermost layers to the outer ones, using Evans Blue dye: living cells are able to exclude the dye (and thus cells remain unstained), whereas dead cells lose membrane integrity and stain blue. At 0 DAG, only the remnants of the cell layers close to the embryo appear stained, and no extensive cell death is observed until well after the mobilization of reserves has finalized. At 42 DAG, cell death has been initiated in the entire tissue.

Tissue death began in the proximal sector of the embryo and extended distally (**Figures 1B,C**). At 0 DAG, the number of fully collapsed cell layers was small. As the degradation of the tissue progressed, the cell walls weakened and lost rigidity, and finally the cells collapsed; the remnants of the degraded cell walls persisted in the crushed cell layers proximal to the embryo (**Figure 1B**). Vacuolar proteins were stained with Amido black at 0 DAG. Storage proteins were relatively scarce and diluted in the water of the central vacuole. Once the germination started, the vacuolar proteins were completely consumed (**Supplementary Figure S1**). During germination, nuclei showed alterations in size and morphology (**Figures 1B,C**): initially large and round, nuclei reduced in size and became fusiform. Also, a progressive increase in chromatin condensation was observed. The reserves were progressively consumed; specifically, starch inside amyloplasts was degraded. Starch depletion began in the proximal sector to the embryo prior to 14 DAG approximately, advancing in the distal direction (**Figure 1B** and **Supplementary Figure S2**). After hydrolysis of the starch, amyloplasts were discharged into the central vacuole (**Figure 1Cb** and **Supplementary Figure S2**). Disorganization of the cytoplasm occurred later with the collapse of the central vacuole, the general degradation of cytoplasmic organelles and plasmolysis (**Figures 1Cb,c**).

The nuclear dismantling was associated with cytoplasmic events during plant PCD. However, chromatin was partially adhered to the nuclear membrane until very advanced the processes of mobilization of reserves (**Figures 1Cd–h**).

### During Storage Mobilization, DNA Fragmentation Accompanied the Progressive Cellular Changes Observed in the Megagametophyte

**FIGURE 2:**
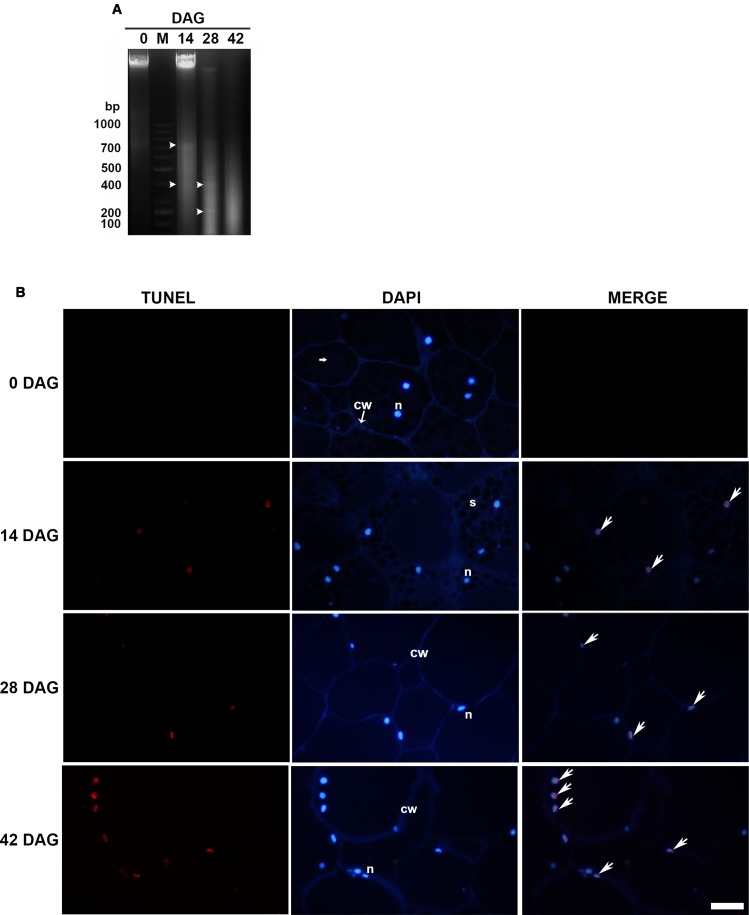
**(A)** Genomic DNA fragmentation assay. Analyses were repeated at least three times on independent biological samples, and representative results are shown. Lane M. 1 kb ladder, Lanes 1, 3, 4, and 5 correspond to 0, 14, 28, and 42 DAG, respectively. Bands are indicated by arrowheads **(B)** TUNEL assay (left column) and DAPI staining (central column) were performed on LRW tissue sections at 0, 14, 28, and 42 DAG. Merged images (right column) confirmed that DAPI co-labeled TUNEL-positive nuclei. Scale bar = 50 μm. Each image is a representative result of observation of at least 5 semi-thin sections of megagametophyte tissue at different DAG.

Analysis of genomic DNA integrity of the total tissue by electrophoresis on agarose gel between 14 and 42 DAG revealed three bands of approximately 700, 400, and 200 bp, respectively. The 700 bp band was clearly weaker between 28 and 42 DAG. Fragmentation was not significant before 14 DAG. Also a faint DNA smearing was observed at 14, 28, and 42 DAG and one well-bound band of high-molecular weight corresponding to intact DNA was visible at 0 and 14 DAG (**Figure 2A**). The lack of detection of a clear laddering could be interpreted as the result of the DNA analysis of a tissue with areas that shown different timing of PCD. To evaluate the *in situ* detection of DNA damage, a TUNEL assay was performed. At 14 DAG, the first TUNEL-positive signals were detected at the innermost layers of the proximal area and later advanced toward the distal area, continuously increasing the number of affected nuclei (**Figure 2B**). Percentage of nuclei labeled was 33, 56, and 92% at 14, 28, and 42 DAG, respectively. Analysis of DAPI-stained nuclei by fluorescence microscopy exhibited the progressive changes in the nuclear morphology, as above-mentioned.

### In the Cells Undergoing PCD, Cys-EP Accumulated in the Cytoplasm and Cell Walls

**FIGURE 3:**
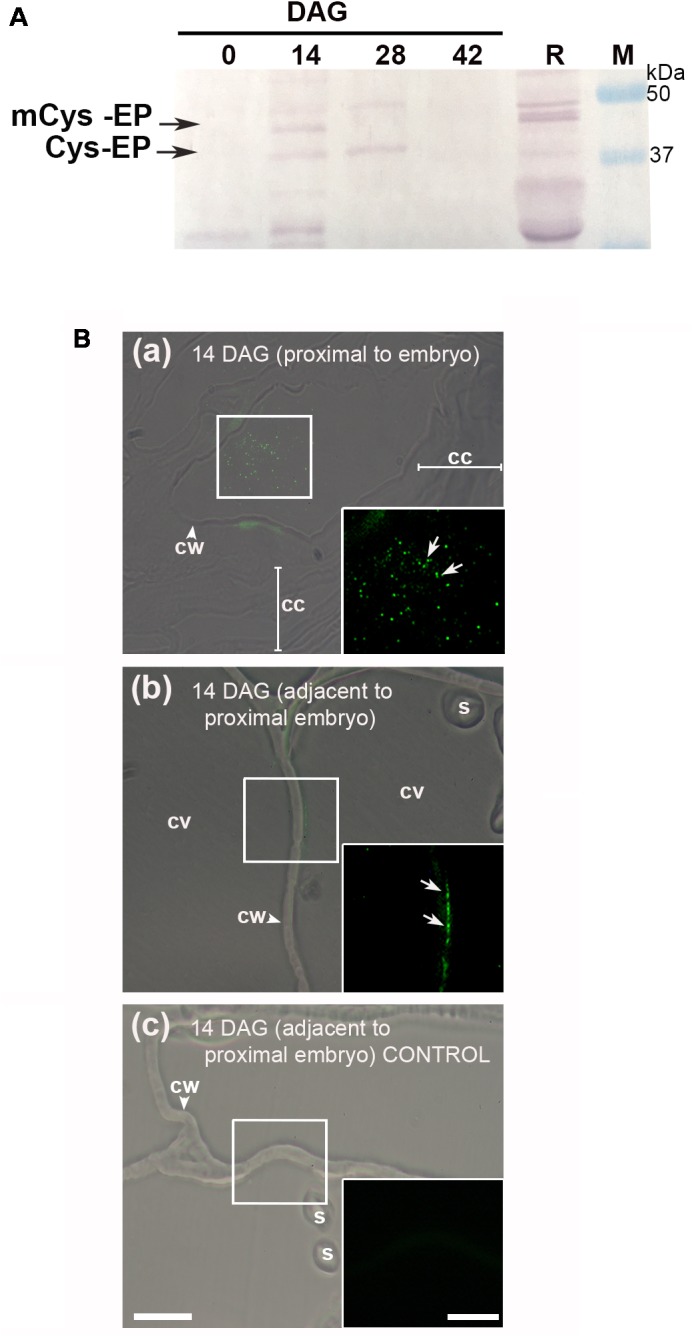
Immunodetection of Cys-EP in *A. angustifolia* megagametophytes, during germination. **(A)** Western blot analysis with anti-RcCys-EP as primary antibody. Proteins from 0, 14, 28, and 42 DAG were separated on a 12% polyacrylamide gel and then transferred to a PVDF membrane. As a control, proteins were extracted from 5 DAG *R. communis* seeds. Analyses were repeated at least three times on independent biological samples, and representative results are shown. **(B)** *In situ* immunolocalization of Cys-EP in megagametophytes of *A. angustifolia* at 14 DAG. Merge bright field and fluorescence imaging of longitudinal sections shows Cys-EP selectively localized to the proximal sector of the embryo **(a)** and adjacent to the proximal sector of the embryo **(b)** at 14 DAG; note labeling in the cytoplasm next to the cell wall **(b)**. **(c)** negative control. Scale bar: **(a–c)** = 20 μm. Inset = 10 μm. Abbreviations: *cc*, crushed cells; *cv*, central vacuole; *cw*, cell wall; s, starch. Each image is a representative result of observation of at least 5 semi-thin sections of a different megagametophyte tissue.

The protein extracts from 0, 14, 28, and 42 DAG were separated by SDS–PAGE and electrophoretically transferred to a PVDF membrane. The western blot clearly showed the presence of the immature and mature forms (approximately 45 and 38 kDa, respectively) of Cys-EP (**Figure 3A**). A major band of 38 kDa, which corresponds to the active form of CysEP, was observed at 14 and 28 DAG. At 42 DAG, neither of the two bands appeared revealed.

The *in situ* accumulation pattern of Cys-EP at 14 DAG was studied on longitudinal sections of the megagametophyte from the proximal and adjacent sectors of the embryo to the proximal cell layers. As mentioned above, the cell walls progressively lost rigidity (**Figure 1B**). **Figure 3B** indicates that, in cells proximal to the embryo, Cys-EP immunolocalized in ricinosomes mixed with vacuolar content and highly vesiculated cytoplasm (a); in vacuolated cells (i.e., with vacuole not collapsed), Cys-EP localized in the parietal cytoplasm, next to the cell wall (b).

### In the Megagametophyte, Zn^2+^ Induced the Activity of Nucleases During Germination

**FIGURE 4:**
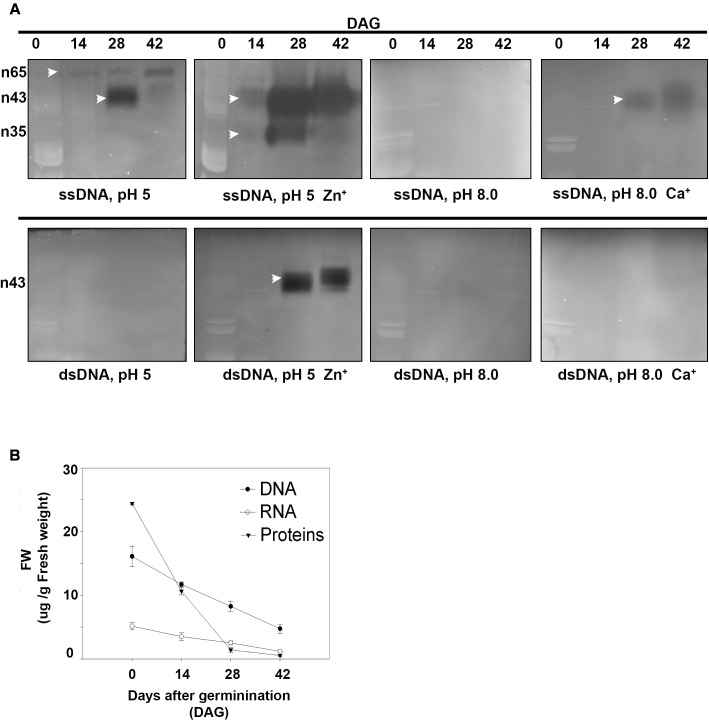
**(A)** Determination of nuclease activity in *A. angustifolia* megagametophytes at 0, 14, 28, and 42 days after germination (DAG) by using the *in-gel* activity assay with ssDNA or dsDNA as substrate. See section “Materials and Methods” for detailed reaction conditions. Analyses were repeated at least three times on independent biological samples, and representative results are shown. **(B)** Changes in soluble protein, DNA and RNA in the megagametophyte, during germination. The three parameters exhibited a marked decrease initiated before 14 DAG. DNA, RNA, and soluble proteins were extracted at 0, 14, 28, and 42 DAG, quantified, and normalized on fresh weight. Data are represented as the mean ± standard error from three independent biological replicates.

Nuclease activity was determined using *in-gel* activity assay with single-stranded (ssDNA) or double-stranded DNA (dsDNA) as substrate at acidic (pH 5) or neutral (pH 8) conditions (**Figure 4**). When ssDNA was used as substrate, the activities of three nucleases with molecular masses of 65, 43, and 35 kDa respectively were detected. The activities of n43 and n35 were strongly enhanced by Zn^2+^ at 28 and 42 DAG. It is worth noting that, although n43 digested ssDNA, in the presence of both Ca^2+^ and Zn^2+^ ions, its ability to digest dsDNA was stimulated only by Zn^2+^ ions at acidic conditions. No bands were obtained when the *in-gel* activity assay was performed at neutral conditions with dsDNA as substrate. The enhancement of Zn^2+^ nuclease activity occurred simultaneously with nuclear DNA fragmentation, i.e., when the band of high molecular weight had disappeared and two bands of low molecular weight (400 and 200 bp, respectively) were markedly visible (**Figure 2A**).

Correlated with the increase in DNA-activity, there was a reduction in both DNA and RNA contents, especially at 14 DAG (**Figure 4B**). During germination, changes were detected in the RNA and soluble protein contents, which, at 42 DAG, reached values close to zero. In addition, the DNA content decreased drastically, reaching values close to 5 μg/g at 42 DAG (i.e., 2.6 times when compared to the control) (**Figure 2A**).

### *In silico* Analysis of Putative Nucleases in *Araucaria angustifolia*

Putative nuclease-encoding genes from *A. angustifolia* were searched within different nucleotide sequence databases. Although no nuclease-encoding genes from *A. angustifolia* were identified, three sequences of transcribed RNAs encoding for putative nucleases from *Araucaria cunninghamii*, a closely related species, were found. These three sequences were obtained from the European Nucleotide Archive^1^ and were submitted under Accession Numbers GCKF01036520.1, GCKF01039343.1, and GCKF01021114.1, respectively. All these sequences were identified from a leaf by transcriptomic analysis, which indicates that they come from genes that are expressed at least in leaf tissue.

**FIGURE 5:**
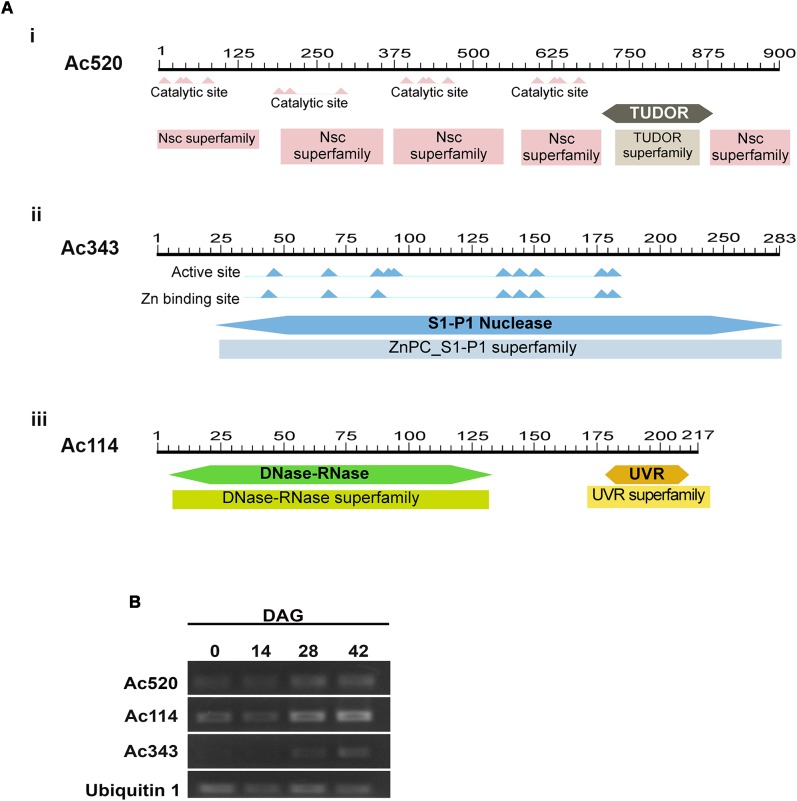
**(A)** *In silico* analysis of putative nuclease in *Araucaria angustifolia.* **(i)**. Representation of the Ac520 protein showing the Staphylococcal nuclease domains at the N terminus and a Tudor domain toward the C terminus. **(ii)** Representation of the Ac343 protein showing the S1-P1 Nuclease domain. **(iii)** Representation of the Ac114 protein showing the DNase-Rnase domain. **(B)** Expression levels of the putative nuclease genes during the germination process in *A. angustifolia*. Amplified products of the Ac520, Ac114, and Ac343 genes by semiquantitative RT-PCR. As a control for constitutive expression, a fragment of the Ubiquitin 1 gene was simultaneously amplified. The results are representative of at least three independent biological replicates.

A sequence analysis of these three putative nuclease-encoding genes was performed using the BLAST tool available at the National Center for Biotechnology Information (NCBI) website (NCBI Resource Coordinators 2016). Firstly, the analysis of the amino acid sequence of the predicted protein encoded by the transcribed RNA of Acc. N° GCKF01036520.1 (herein after referred to as putative protein Ac520) revealed high similarity to several Tudor Staphylococcal nucleases (Tudor-SN). The sequence with the highest similarity to Ac520 was a Tudor-SN from *Wollemia nobilis* (Uniprot Acc. N° A0A0C9RQ39), sharing 98.4% of identical amino acids. Ac520 also showed 81% identity to a Tudor-SN from *Picea abies* (Uniprot Acc. No. Q0JRI3), and 64.5, 64.4, and 63.5% identity to ribonuclease TUDOR1 (Uniprot Acc. No. Q8VZG7), isoform 2 of ribonuclease TUDOR 1 (Uniprot Acc. No. Q8VZG7-2) and ribonuclease TUDOR 2 (Uniprot Acc. No. Q9FLT0) from *Arabidopsis thaliana*, respectively. Furthermore, Ac520 has the conserved domains of Tudor-SN nucleases (**Figure 5Ai**), that is, four SN domains at the N-terminus and a Tudor domain at the C-terminus (Liu et al., 2010). Tudor-SN proteins have been shown to be involved in the control of seed germination in *A. thaliana* (Liu et al., 2010) and in the regulation of PCD in plants (Sundström et al., 2009; Coll et al., 2010; Reape and McCabe, 2010; Tsiatsiani et al., 2011). The analysis of the amino acid sequence of the predicted protein encoded by the transcribed RNA of Acc. No. GCKF01039343.1 (herein after referred to as putative protein Ac343) showed the presence of a S1-P1 nuclease domain (**Figure 5Aii**). Ac343 showed 63.1 and 60.8% identity to ENDO4 (Uniprot Acc. No. F4JJL0) and ENDO2 (Uniprot Acc. No. Q9C9G4) from *A. thaliana*, respectively. This last nuclease was involved in RNA, ssDNA, and dsDNA degradation, with a preference for ssDNA and RNA (Ko et al., 2012). Finally, the analysis of the amino acid sequence of the predicted protein encoded by the transcribed RNA of Acc. No. GCKF01021114.1 (herein after referred to as putative protein Ac114) revealed the presence of a conserved DNase-RNase domain (**Figure 5Aiii**). This domain is characteristic of a family of bifunctional nucleases having both DNase and RNase activity. In fact, Ac114 shares 80% of amino acid identity with a predicted bifunctional nuclease from *Picea sitchensis* (Uniprot Acc. No. A9NUL3) previously reported by Ralph et al. (2008). Ac114 also showed 67.6% identity to BBD2 (Uniprot Acc. No. Q93VH2) and 63.7% identity to BBD1 (Uniprot Acc. No. Q9FWS6) from *A. thaliana*.

### Expression Levels of Putative Nuclease Genes During Germination in Megagametophytes of *Araucaria angustifolia*

To evaluate the expression of genes from *A. angustifolia* encoding for orthologous nucleases of Ac520, Ac343, and Ac114, primers were designed to carry out semi-quantitative RT-PCR. It should be noted that *A. angustifolia* and *A. cunninghamii* are very closely related species and therefore the orthologous sequences are not expected to have significant differences. The transcript levels of the genes encoding Ac520, Ac343, and Ac114 were analyzed in the megagametophyte of *A. angustifolia* at 0, 14, and 28 years 42 DAG (**Figure 5B**). Expression of the Ac520 and Ac114 genes increased along the germination process, with higher expression levels at 28 and 42 DAG, whereas that of the Ac343 gene was only detected at 28 and 42 DAG.

## Discussion

Nuclease activation, DNA fragmentation and reserve mobilization in the megagametophyte of *Araucaria angustifolia* occurred simultaneously during the first 4 weeks following germination. During storage mobilization, DNA fragmentation accompanied the progressive cellular changes observed in the cells of the megagametophyte. On the basis of these results, we propose that the pathway of cell death in the *A. angustifolia* megagametophyte is PCD. As mentioned, to date, the dismantling of the megagametophyte during germination has been studied in only two species of Gymnosperms: *Araucaria bidwillii* and *Picea glauca*. In *A. bidwillii*, a species that is very closely related to *A. angustifolia* and also produces starchy seeds, cell death has been reported to occur through necrosis and probably also PCD in some cells (Casani et al., 2009). In *Picea glauca*, the megagametophyte is a tissue whose cells store proteins and lipids and lacks vacuoles, and the reserve mobilization pattern and the PCD pathway are similar to those described in the aleurone layer of cereals or in the endosperm of tomato (He and Kermode, 2003).

Autophagy is a process known to mediate the degradation of residual proteins and aggregates of insoluble proteins and lipids, and to remove damaged organelles (Levine and Klionsky, 2004; Mizushima, 2007; Rodriguez-Navarro and Cuervo, 2010). In addition, autophagic assimilation and reprocessing can maintain cellular homeostasis, responding to environmental changes, but can also function in association with the PCD process (Kourtis and Tavernarakis, 2009; Kuma and Mizushima, 2010). Although it is known that autophagy also mediates bulk degradation of the cytosol and organelles in plants, its role in plastid catabolism is largely unknown (Wada et al., 2008). In the megagametophyte of *A. angustifolia*, we observed that, after starch hydrolysis, autophagy was responsible for the final degradation of amyloplasts. This process seems to be similar to that occurring in chloroplasts of *Arabidopsis* leaves during senescence (Wada et al., 2008), although this issue needs further investigation.

KDEL-tailed Cys-EPs digest extensin, thus supporting the final cell collapse during PCD. Cys-EPs were detected for the first time in the endosperm of *Ricinus communis* (Schmid et al., 2001) and the epigeal cotyledons of *Vigna mungo* (Toyooka et al., 2001) during germination. Cys-EPs have also been identified in the megagametophyte of *Picea glauca* (He and Kermode, 2003). Here, we detected a Cys-EP in the megagametophyte of *A. angustifolia* during germination by using an antibody raised against a Cys-EP purified from ricinosomes of the endosperm of *Ricinus communis* seeds during germination. This Cys-EP was immunolocalized in ricinosomes mixed with vacuolar content and in the parietal cytoplasm, next to the cell wall. By western blot, we recognized both the proform and mature form of the enzyme at 14 and 28 DAG but not at 42 DAG. According to Schmid et al. (1998), the other bands that we detected correspond to a precursor protease, a C-terminally truncated active form and to degradation products.

In the present study, two Zn^2+^-dependent nucleases of 35 and 43 kDa were induced at 28–42 DAG at acid pH. Nucleases of the S1/P1 family are thought to be similar to nucleases type I, showing maximal activity at acidic pH and Zn^2+^ dependency (Sugiyama et al., 2000).

Here, we found three sequences of transcribed RNAs, Ac520, Ac343, and Ac114, encoding for putative nucleases. Ac520 revealed high similarity to several Tudor-SN, with highest similarity to a Tudor-SN from *Wollemia nobilis* (Uniprot Acc. No. A0A0C9RQ39), with which it shared 98.4% of identical amino acids. *Wollemia nobilis* is the only species of the genus *Wollemia* that also belongs to the family Araucariaceae (Division Pinophyta-Order Pinales) (Jones et al., 1995). According to Setoguchi et al. (1998), the Araucariaceae are well defined by the rbcL sequence, and their monophyly is supported by a bootstrap value of 100%.

Likewise, Ac520 showed 81% identity to a Tudor-SN from *Picea abies* (Uniprot Acc. No. Q0JRI3), a species of the family Pinaceae, which also belongs to the Division Pinophyta-Order Pinales; this species is phylogenetically and temporally very distant from *Araucaria*. In fact, the Pinaceae diverged from the lineage ultimately leading to *Araucaria* in the Late Carboniferous to Early Permian periods, approximately 300–250 million years ago (Gernandt et al., 2008; Leslie et al., 2012). Ac520 also exhibited identity to TUDOR ribonucleases (Uniprot Acc. No. Q8VZG7, Uniprot Acc. No. Q8VZG7-2, Uniprot Acc. No. Q9FLT0) from *Arabidopsis thaliana*.

It is important to note that *A. thaliana* is an Angiosperm species phylogenetically very distant from *Araucaria*. Furthermore, Ac520 has the conserved domains of Tudor-SN, that is, four SN domains at the N-terminus and a Tudor domain at the C-terminus (Liu et al., 2010). Tudor-SN proteins have been shown to be involved in the control of seed germination in *A. thaliana* (Liu et al., 2010) and in the regulation of PCD in plants (Sundström et al., 2009; Coll et al., 2010; Reape and McCabe, 2010; Tsiatsiani et al., 2011). Ac343 showed the presence of a S1-P1 nuclease domain, and 63.1 and 60.8% identity to ENDO 4 (Uniprot Acc. No. F4JJL0) and ENDO2 (Uniprot Acc. No. Q9C9G4) from *Arabidopsis thaliana*, respectively. This last nuclease is involved in RNA, ssDNA, and dsDNA degradation, with a preference for ssDNA and RNA (Ko et al., 2012). Ac343 also presented 55% amino acid identity to the best characterized plant S1-like nucleases, ZEN1 of *Zinnia elegans* and *Arabidopsis* ENDO1 (also named bifunctional nuclease1, BFN1). Previous reports have demonstrated that BFN1 and ZEN1 are involved in different forms of PCD (Pérez-Amador et al., 2000; Ko et al., 2012; Lesniewicz et al., 2013). Finally, Ac114 revealed the presence of a conserved DNase-RNase domain, which is characteristic of a family of bifunctional nucleases having both DNase and RNase activity. In fact, Ac114 shared 80% of amino acid identity with a predicted bifunctional nuclease from *Picea sitchensis*, a species of the family Pinaceae (Uniprot Acc. No. A9NUL3) previously reported by Ralph et al. (2008). Ac114 also showed 67.6% identity to BBD2 (Uniprot Acc. No. Q93VH2) and 63.7% identity to BBD1 (Uniprot Acc. No. Q9FWS6) from *A. thaliana.*

The results of the present study add knowledge on the dismantling of the megagametophyte of mature starchy seeds in species of the genus *Araucaria*, a storage tissue that stores starch as the main reserve substance, as well as on the PCD pathway, by revealing new insights into the role of nucleases and the expression patterns of putative nuclease genes during germination.

## Author Contributions

SM and ML-F conceived, designed and coordinated the project, and initiated the project. ML-F coordinated the field work and sampling. LM, MC, FR, LF, and ML-F performed laboratory work. LM, MC, FR, LF, SM, and ML-F performed the data analysis. SM and ML-F wrote the first draft of the paper. All authors contributed to discussing the results and editing the paper.

## Conflict of Interest Statement

The authors declare that the research was conducted in the absence of any commercial or financial relationships that could be construed as a potential conflict of interest.

## Footnotes

1 https://www.ebi.ac.uk/ena

## Supplementary Material

FIGURE S1 Amido black staining at 0, 14, and 42 DAG in the megagametophyte sections. Amido black stains total proteins. They were visible at 0 DAG. At 14 and 42 DAG, vacuoles shown that storage proteins are consumed early. In all sections, nuclear proteins were also dyed. Abbreviations: *cc*, crushed cells; *v*, vacuole; *cw*, cell wall; am, amyloplasts. Scale bar = 50 μm. Each image is a representative result of observation of at least 30 semi-thin sections of a different megagametophyte tissue at different DAG.

FIGURE S2 Lugol staining at 0, 14, and 42 DAG in the megagametophyte sections. Starch was progressively consumed during germination. Starch was histochemically identified by lugol staining. Vacuolar transport of entire amyloplast can be observed (arrows). Amyloplasts finished being dismantled and starch totally consumed within the central vacuole. Abbreviations: *cc*, crushed cells; *v*, vacuole; *cw*, cell wall; am, amyloplasts. Scale bar = 50 μm. Each image is a representative result of observation of at least 30 semi-thin sections of a different megagametophyte tissue at different DAG.
