# Supplementary figures and images for: Activation of Nucleases, PCD, and Mobilization of Reserves in the Araucaria angustifolia Megagametophyte During Germination
Source: Front Plant Sci. 2018 Aug 30;9:1275. doi: 10.3389/fpls.2018.01275 (PMC6125354; doi:10.3389/fpls.2018.01275)

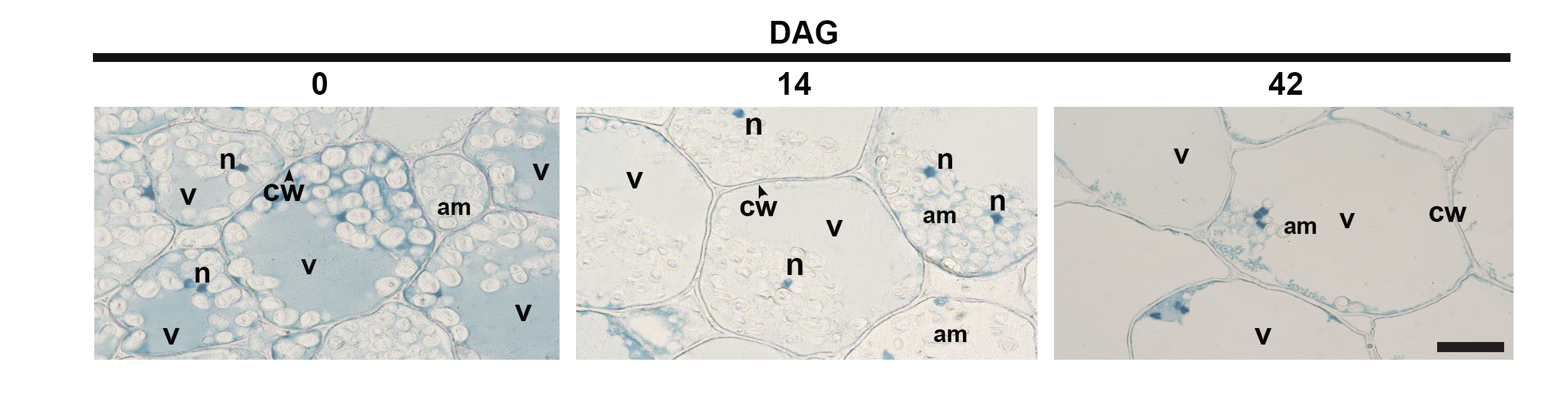

Supplement: FIGURE S1 — Amido black staining at 0, 14, and 42 DAG in the megagametophyte sections. Amido black stains total proteins. They were visible at 0 DAG. At 14 and 42 DAG, vacuoles shown that storage proteins are consumed early. In all sections, nuclear proteins were also dyed. Abbreviations: cc, crushed cells; v, vacuole; cw, cell wall; am, amyloplasts. Scale bar = 50 μm. Each image is a representative result of observation of at least 30 semi-thin sections of a different megagametophyte tissue at different DAG. [file Image_1.TIF]

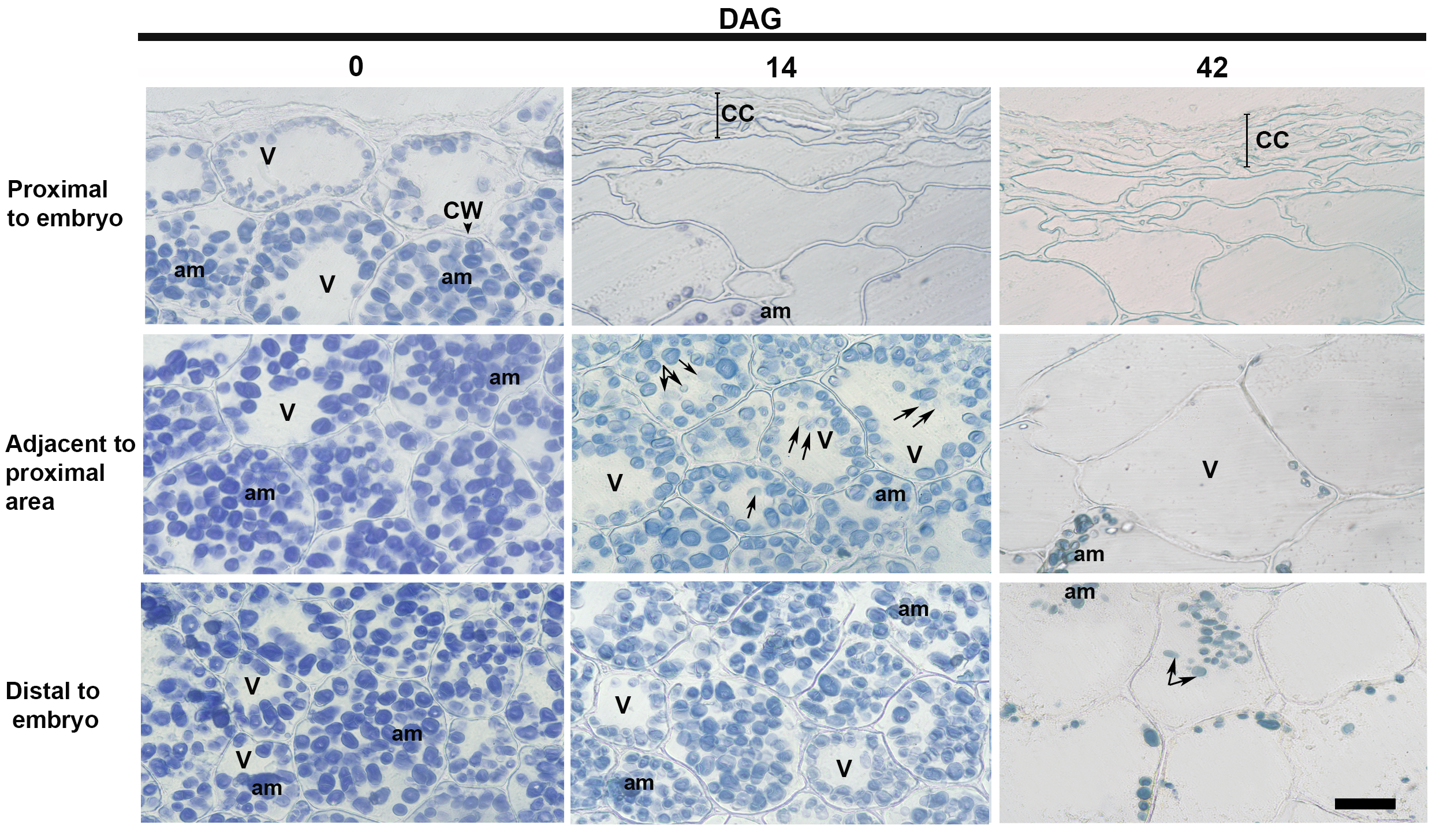

Supplement: FIGURE S2 — Lugol staining at 0, 14, and 42 DAG in the megagametophyte sections. Starch was progressively consumed during germination. Starch was histochemically identified by lugol staining. Vacuolar transport of entire amyloplast can be observed (arrows). Amyloplasts finished being dismantled and starch totally consumed within the central vacuole. Abbreviations: cc, crushed cells; v, vacuole; cw, cell wall; am, amyloplasts. Scale bar = 50 μm. Each image is a representative result of observation of at least 30 semi-thin sections of a different megagametophyte tissue at different DAG. [file Image_2.TIF]
